# Reduced Age-Related Gray Matter Loss in the Orbitofrontal Cortex in Long-Term Meditators

**DOI:** 10.3390/brainsci13121677

**Published:** 2023-12-06

**Authors:** Florian Kurth, Sarah Strohmaier, Eileen Luders

**Affiliations:** 1 School of Psychology, University of Auckland, Auckland 1010, New Zealand; 2 Psychology Discipline, Institute for Health and Sport, Victoria University, Melbourne, VIC 3011, Australia; 3 Department of Women’s and Children’s Health, Uppsala University, 751 85 Uppsala, Sweden; 4 Laboratory of Neuro Imaging, School of Medicine, University of Southern California, Los Angeles, CA 90033, USA

**Keywords:** age, brain, gray matter, meditation, mindfulness, orbitofrontal cortex

## Abstract

The orbitofrontal cortex (OFC) is a functionally heterogeneous brain region contributing to mental processes relating to meditation practices. The OFC has been reported to decline in volume with increasing age and differs in volume between meditation practitioners and non-practitioners. We hypothesized that the age-related decline of the OFC is diminished in meditation practitioners. We tested this hypothesis in a sample of 50 long-term meditators and 50 matched controls by correlating chronological age with regional gray matter volumes of the left and right OFC, as well as in seven left and right cytoarchitectonically defined subregions of the OFC (Fo1–Fo7). In both meditators and controls, we observed a negative relationship between age and OFC (sub)volumes, indicating that older participants have smaller OFC volumes. However, in meditators, the age-related decline was less steep compared to controls. These age-related differences reached significance for left and right Fo2, Fo3, Fo4, and Fo7, as well as left Fo5 and right Fo6. Since different subregions of the OFC are associated with distinct brain functions, further investigations are required to explore the functional implications of these findings in the context of meditation and the aging brain.

## 1. Introduction

Meditation research has grown exponentially in recent years, and an increasing number of studies suggest that meditation might slow age-related cognitive decline as well as tissue loss in the brain [1,2,3,4,5,6,7,8,9,10]. One brain region reported to be involved in meditation and altered in meditation practitioners is the orbitofrontal cortex [7,11,12,13,14]. Since the orbitofrontal cortex is also known to decrease in volume and thickness with age [15,16,17,18,19,20,21], age-related decline in the orbitofrontal cortex may be diminished in meditation practitioners.

The current study set out to investigate the possible effects of meditation on age-related gray matter volume loss in the orbitofrontal cortex within a sample of 50 long-term meditators and 50 age- and sex-matched controls. To assess anatomically and functionally meaningful orbitofrontal subareas, we used the cytoarchitectonic parcellation of this region into medial orbitofrontal areas Fo1–Fo3 [22] and lateral orbitofrontal areas Fo4–Fo7 [23] in addition to the overall orbitofrontal cortex, which was defined as the composite of these subareas (OFC = Fo1 + Fo2 + Fo3 + Fo4 + Fo5 + Fo6 + Fo7). Gray matter volume for each area was measured using an advanced region-of-interest technique, combining image-based signal intensities and cytoarchitectonically defined probabilities [24,25,26]. In both meditators and controls, we anticipated finding a negative relationship between age and gray matter volume. Nevertheless, our hypothesis was that the age-related reduction in volume would be more moderate in long-term meditators compared to controls. Furthermore, we expected that the magnitude of these effects would vary across the subareas of the orbitofrontal cortex.

## 2. Materials and Methods

### 2.1. Study Sample and Brain Images

The study included 50 meditation practitioners and 50 control subjects, with ages between 24 and 77 years; a detailed sample description is provided elsewhere [9]. Importantly, both groups were matched for sex (28 men and 22 women in each group) as well as for age (mean ± SD controls: 51.4 ± 12.8 years; meditators: 50.4 ± 11.8 years). In addition, both groups were comparable in terms of handedness and education, and were free from neurological and psychiatric disorders, as previously described [27]. The meditators had an active lifetime practice between 4 and 46 years (mean ± SD: 19.8 ± 11.4 years); an overview of individual meditation practices in this sample has been provided elsewhere [9]. All study participants gave informed consent in accordance with the policies and procedures of the Institutional Review Board at the University of California (UCLA).

Brain scans for the control group were sourced from the International Consortium for Brain Mapping (ICBM) database, which contains data from healthy adults (https://ida.loni.usc.edu/). Brain scans for the meditators were collected at the University of California, Los Angeles (UCLA) using the same 1.5 Tesla Siemens Sonata scanner and the same scanning parameters employed for the control group: a T1-weighted magnetization-prepared rapid acquisition gradient echo (MPRAGE) sequence with a 4.38 ms echo time, a 1900 ms repetition time, a 15° flip angle, 160 contiguous sagittal slices, a 256 × 256 mm^2^ field-of-view, and a 1 × 1 × 1 mm^3^ voxel size.

### 2.2. Data Processing and Volume Extraction

All brain images were processed and analyzed in Matlab (https://www.mathworks.com/products/matlab.html) using SPM12 (https://www.fil.ion.ucl.ac.uk/spm/software/spm12), the CAT12 toolbox [28], and the Julich-Brain Atlas [29,30], as described elsewhere [24,25,26,31]. After applying corrections for magnetic field inhomogeneities, brain images were segmented into gray matter, white matter, and cerebrospinal fluid. Subsequently, the gray matter segments were spatially normalized to the Shooting template [32] and modulated to preserve the original voxel-wise gray matter [26,33,34]. Total intracranial volume (TIV) was calculated in native space and later included as a covariate in the statistical model.

Region-specific gray matter volumes of the orbitofrontal subregions Fo1–Fo7 [22,23] were calculated using the cytoarchitectonic probability maps provided with the Julich-Brain Atlas [29,30]. More specifically, in a first step, the probability maps were spatially normalized to the Shooting template to ensure accurate spatial correspondence with the modulated normalized gray matter segments (see previous section). This was followed by voxel-wise multiplication of these normalized probability maps (i.e., left and right Fo1-Fo7) with the normalized and modulated gray matter segments. The resulting voxel-wise measures were multiplied by the voxel volume and added up to estimate region-specific gray matter volumes (in mm^3^), as detailed elsewhere [26]. In addition, the volume for the orbitofrontal cortex as a whole was calculated separately for each hemisphere (OFC = Fo1 + Fo2 + Fo3 + Fo4 + Fo5 + Fo6 + Fo7).

### 2.3. Statistical Analysis

Applying a mass-univariate general linear model, we designated the volumes of the left and right regions of interest (OFC and Fo1–Fo7) as dependent variables, with group, age, and the group-by-age interaction as independent variables. Age was centered at 50 years. Sex and TIV were treated as nuisance variables. As assumptions for parametric testing were not met in all cases (the residuals for left Fo1 and left Fo7 were not normally distributed as determined using a Lilliefors test), significance was established for all regions using a Monte Carlo simulation with 10,000 permutations using the Smith procedure [35,36]. Results were corrected for multiple comparisons by controlling the false discovery rate [37,38]. Significant group-by-age interactions were followed by conducting post hoc tests investigating age-related correlations within meditators and controls separately. For this, we applied multiple regression models, where sex and TIV were treated again as nuisance variables. Last but not least, the region-specific annual volume loss (in %) was calculated at age 50 using the beta-estimates of the statistical model [5,24].

## 3. Results

As detailed in Table 1, there was a significant group-by-age interaction for the left and right orbitofrontal cortex overall (OFC), as well as for left and right Fo2, Fo3, Fo4, and Fo7. In addition, significant group-by-age interactions were observed for left Fo5 and right Fo6. Effect sizes (calculated as Cohen’s d) ranged between d = 0.374 and d = 0.806, suggesting a range from small to large effects.

**Figure 1:**
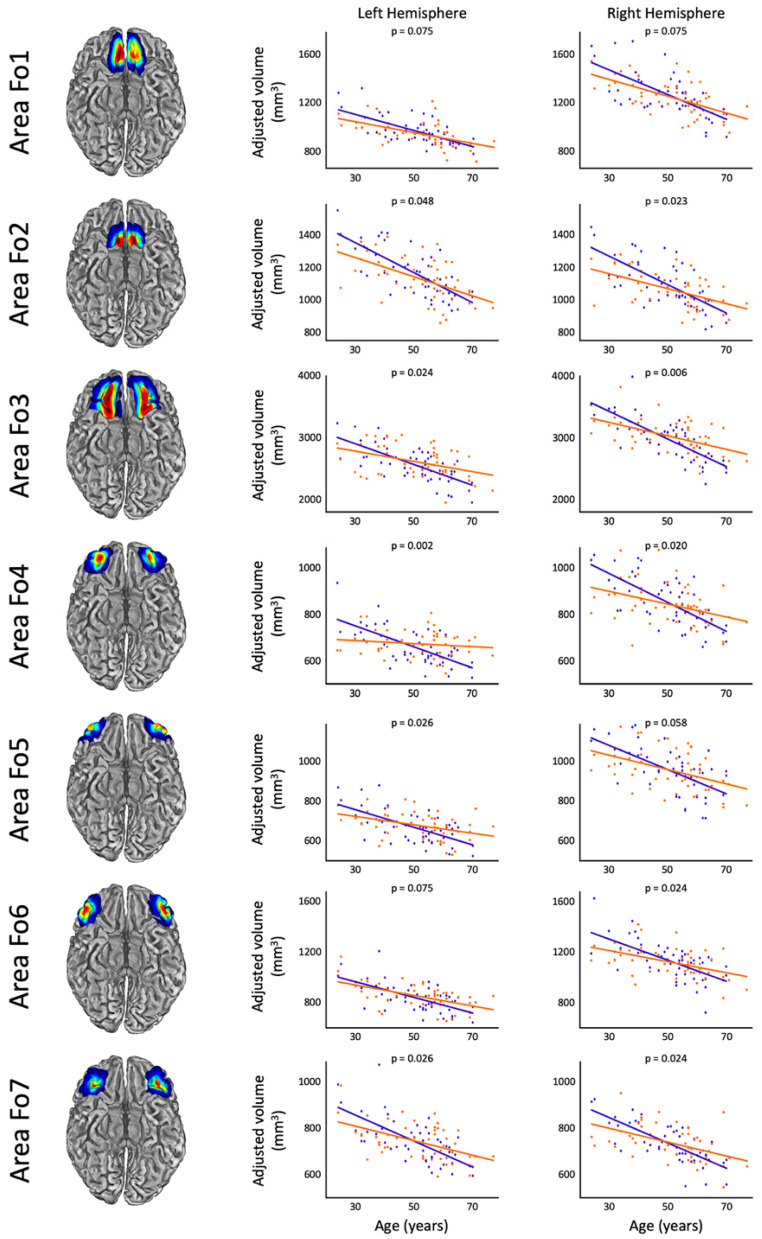
Links between age and orbitofrontal gray matter. Left: probability maps of orbitofrontal areas (Fo1–Fo7) based on 10 post-mortem brains [24,25]. The colors indicate the region-specific probability (blue = 25%, green = 50%, orange = 75%, red = 100%). Right: scatterplots depict the correlations between age and the region-specific gray matter volumes in meditators (orange) and controls (blue). Values are adjusted for sex and TIV as per the statistical model.

Group-specific correlations between age and orbitofrontal gray matter are visualized in Figure 1. As shown in Table 2, they were exclusively negative both in meditators and controls (i.e., the older the individual, the smaller the volumes). All of these negative associations were significant in controls. They were also significant in meditators with the exception of left Fo4. However, the slopes of the regression trajectories were notably steeper in controls compared to meditators, and the correlation coefficients were mostly higher in controls. The rates of annual tissue loss at age 50 ranged between 0.6% and 0.8% in controls and between 0.1% and 0.6% in meditators (see Table 2).

## 4. Discussion

Effects of aging on the orbitofrontal cortex were observed in both groups, with older participants having smaller volumes of the orbitofrontal cortex. However, aging trajectories were less steep in meditators compared to controls. These results thus support our hypothesis of diminished age-related gray matter loss in the orbitofrontal cortex in long-term meditation practitioners.

These findings seem to be in agreement with outcomes of previous studies that detected larger gray matter volumes [7,11,14] as well as increased glucose metabolism [7] in the orbitofrontal cortex in meditators compared to controls. They also seem to be aligned with reports of increased orbitofrontal activity during meditation [12]. The orbitofrontal cortex is involved in processing and regulating pleasant and unpleasant emotions; it is also implicated in reward-related learning, language, working memory, and memory in general [22,23,39,40,41,42,43,44,45,46,47]. Thus, the outcomes of our study also seem to corroborate other research suggesting that regular mindfulness practices are linked to improved emotion regulation [48,49], increased empathy [50], as well as enhanced memory, attention, and language processing [51,52].

With respect to age-related brain preservation, the findings from the current study suggest that continuous long-term meditation practice has the potential to slow down age-related volume loss within the orbitofrontal cortex and its subregions. This may be a by-product of the constant training and resulting neuroplastic changes known to increase local tissue volumes [10,53,54,55]. However, other mechanisms, such as meditation-induced reduction of stress responses and inflammation, might be at play as well (for a review, see [10]). In further support of this assumption, previous studies in both humans and animals revealed that adverse life events and chronic stress are linked to a reduction of gray matter volumes or specifically GABAergic neurons within the orbitofrontal cortex [56,57].

Further research is clearly necessary to replicate the observed effects in larger samples, ideally using longitudinal and randomized controlled designs over an extended period of time. The latter will also help resolve whether the orbitofrontal cortex of experienced meditators was already different prior to their meditation practice and how much of the age-related effect was a consequence of meditation practice rather than a pre-existing characteristic. Follow-up studies might also want to collect measures of stress and/or glucocorticoid levels to assess the effects of (meditation-induced) stress reduction on orbitofrontal regions. Similarly, measures of anxiety and depression, as well as health and lifestyle indicators, might yield valuable information. Last but not least, given that the different subregions of the orbitofrontal cortex serve different functions, future studies may significantly enhance this field of research by including functional and behavioral measures to determine links between brain structure, brain function, and behavior.

## Figures and Tables

**Table 1:** Group-by-age interactions.

Area	Left Hemisphere			Right Hemisphere		
	Effect Size (Cohen’s d)	Significance (t)	Significance (*p*, FDR-Corrected)	Effect Size (Cohen’s d)	Significance (t)	Significance, (*p*, FDR-Corrected)
OFC	0.644	3.123	0.004 *	0.663	3.212	0.004 *
Fo1	0.304	1.473	0.075	0.319	1.545	0.075
Fo2	0.374	1.814	0.048 *	0.513	2.488	0.023 *
Fo3	0.470	2.276	0.024 *	0.628	3.044	0.006 *
Fo4	0.806	3.909	0.002 *	0.525	2.547	0.020 *
Fo5	0.439	2.130	0.026 *	0.331	1.603	0.058 ^T^
Fo6	0.284	1.376	0.075	0.441	2.138	0.024 *
Fo7	0.433	2.097	0.026 *	0.480	2.325	0.024 *

OFC = Orbitofrontal cortex (composite of subareas Fo1-7); FDR = false discovery rate. * Significant; ^T^ trend for significance (*p*-value, uncorrected = 0.047).

**Table 2:** Age-related links and annual volume loss at age 50, separately within meditators and controls.

	Area	Meditators			Controls		
		Correlation Coefficient (r)	Significance (*p*, FDR-Corrected)	Volume Loss (%)	Correlation Coefficient (r)	Significance (*p*, FDR-Corrected)	Volume Loss (%)
Left	OFC	−0.498	<0.001 *	−0.376	−0.674	<0.001 *	−0.704
	Fo1	−0.434	<0.001 *	−0.477	−0.522	<0.001 *	−0.696
	Fo2	−0.451	<0.001 *	−0.514	−0.561	<0.001 *	−0.787
	Fo3	−0.330	0.001 *	−0.318	−0.519	<0.001 *	−0.656
	Fo4	−0.105	0.153	−0.098	−0.526	<0.001 *	−0.686
	Fo5	−0.294	0.003 *	−0.313	−0.483	<0.001 *	−0.667
	Fo6	−0.409	<0.001 *	−0.489	−0.495	<0.001 *	−0.740
	Fo7	−0.386	0.001 *	−0.421	−0.540	<0.001 *	−0.756
Right	OFC	−0.506	<0.001 *	−0.403	−0.683	<0.001 *	−0.751
	Fo1	−0.461	<0.001 *	−0.559	−0.547	<0.001 *	−0.812
	Fo2	−0.399	<0.001 *	−0.431	−0.578	<0.001 *	−0.801
	Fo3	−0.416	<0.001 *	−0.362	−0.626	<0.001 *	−0.754
	Fo4	−0.305	0.003 *	−0.329	−0.526	<0.001 *	−0.734
	Fo5	−0.345	0.001 *	−0.379	−0.469	<0.001 *	−0.640
	Fo6	−0.347	0.001 *	−0.391	−0.518	<0.001 *	−0.739
	Fo7	−0.407	<0.001 *	−0.407	−0.571	<0.001 *	−0.745

FDR = false discovery rate. * Significant.

## Data Availability

While the conditions of our ethics approval do not permit public archiving of anonymized study data for the meditation sample, the data for the control sample are available via the IDA database at the Laboratory of Neuro Imaging (https://ida.loni.usc.edu/).
